# Novel Mutation in a Patient with Cholesterol Ester Storage Disease

**DOI:** 10.1155/2015/347342

**Published:** 2015-02-05

**Authors:** Patrick Lin, Sheela Raikar, Jennifer Jimenez, Katrina Conard, Katryn N. Furuya

**Affiliations:** ^1^Department of Pediatrics, Nemours/Alfred I. duPont Hospital for Children, Wilmington, DE 19803, USA; ^2^Thomas Jefferson University, Philadelphia, PA 19107, USA; ^3^Division of Pediatric Gastroenterology, Hepatology, and Nutrition, Nemours/Alfred I. duPont Hospital for Children, Wilmington, DE 19803, USA; ^4^Department of Clinical and Anatomic Pathology, Nemours/Alfred I. duPont Hospital for Children, Wilmington, DE 19803, USA

## Abstract

Cholesterol ester storage disease (CESD) is a chronic liver disease that typically presents with hepatomegaly. It is characterized by hypercholesterolemia, hypertriglyceridemia, high-density lipoprotein deficiency, and abnormal lipid deposition within multiple organs. It is an autosomal recessive disease that is due to a deficiency in lysosomal acid lipase (LAL) activity, which is coded by the lysosomal acid lipase gene (LIPA). We describe the case of a 5-year-old south Asian female incidentally found to have hepatomegaly, and subsequent workup confirmed the diagnosis of CESD. DNA sequencing confirmed the presence of a novel hepatic mutation. It is a four-nucleotide deletion c.57_60delTGAG in exon 2 of the LIPA gene. This mutation is predicted to result in a premature translation stop downstream of the deletion (p.E20fs) and, therefore, is felt to be a disease-causing mutation.

## 1. Introduction

Cholesterol ester storage disease (CESD) is a chronic liver disease that typically presents with hepatomegaly. It is associated with hypercholesterolemia, hypertriglyceridemia, high-density lipoprotein (HDL) deficiency, and abnormal lipid deposition within multiple organs [1]. It is an autosomal recessive disease that is due to a deficiency in lysosomal acid lipase (LAL) activity. LAL hydrolyzes cholesterol esters and triglycerides within the lysosomes of hepatocytes. In the absence of LAL, lipid builds up in the endoplasmic reticulum of hepatocytes. This leads to the development of hepatic steatosis with the eventual development of fibrosis and micronodular cirrhosis [2, 3].

Disease-causing mutations in the LAL gene (lysosomal acid lipase gene [LIPA]) may result in the clinical presentation of CESD or Wolman disease (WD). Wolman disease is a severe, early-onset presentation caused by a mutation in the LIPA gene that results in the absence of LAL activity [2]. It is typically fatal within the first few months of life. On the other hand, CESD is more prevalent than WD and typically presents later in life. We present a patient with CESD who has a novel disease-causing mutation in the LIPA gene.

## 2. Case Presentation

A 5-year-old south Asian female was incidentally found to have hepatomegaly on a trip to India, where she became acutely ill with fever, vomiting, and abdominal pain. Blood work done at that time demonstrated mildly elevated liver function tests (AST 48 U/L, ALT 80 U/L), and an abdominal ultrasound revealed hepatomegaly without biliary or splenic abnormalities. She was prescribed an antibiotic and her symptoms gradually resolved.

**Figure 1:**
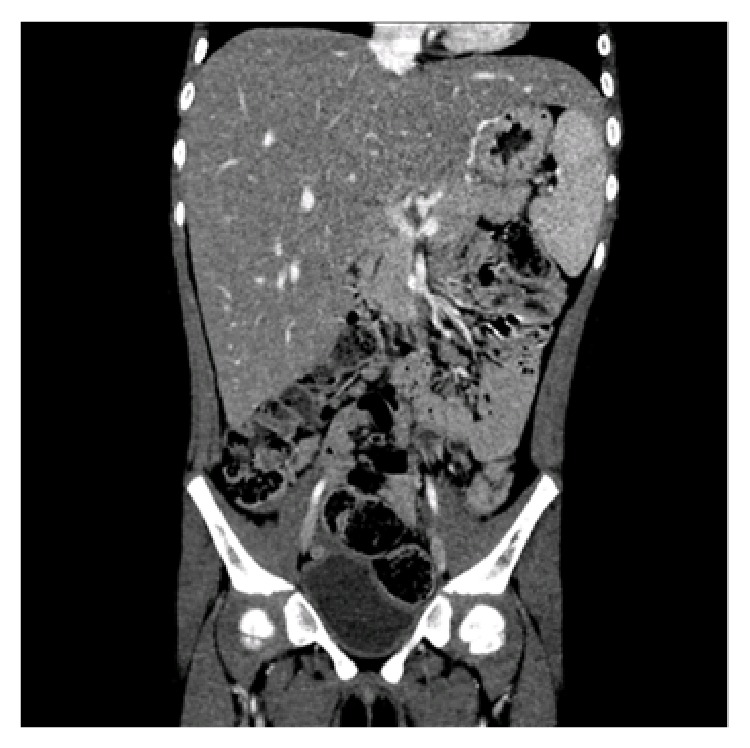
Computed tomography of abdomen and pelvis. The liver is enlarged, measuring up to 17 cm in width and craniocaudal dimension. The contour is smooth. The hepatic parenchyma is homogenous without a focal mass. There is no intrahepatic biliary dilatation. The portal and hepatic venous systems are patent and nondilated.

A month after her return to the United States, her pediatrician noted persistent hepatomegaly and the new development of fever. She was referred to the emergency department because of suspicion of a tropical infectious disease. A repeat ultrasound demonstrated persistent hepatomegaly. Her transaminases (AST 133 U/L, ALT 112 U/L) remained elevated. The infectious workup was negative for cytomegalovirus, hepatitis A, Lyme disease, and malaria. She did have a positive Epstein-Barr virus IgM. Repeat blood work was obtained at follow-up infectious disease clinic visit where she was again found to have hepatomegaly (7 cm below the costal margin) and mildly abnormal transaminases. A computed tomography of the abdomen and pelvis (Figure 1) was performed that demonstrated hepatomegaly but had otherwise normal findings. Her fever resolved within 2 weeks. She was otherwise well and was not on any medications. There was no family history of liver disease. Her immunizations were up to date. Because of persistent hepatomegaly, she was referred to the liver clinic.

In the liver clinic, she was noted to have an enlarged, firm liver, palpable 8 cm below the costal margin with no associated splenomegaly. Test results for autoimmune hepatitis, WD, and alpha-1-antitrypsin deficiency were negative. Nonfasting lipid levels were abnormal; repeat testing showed a fasting total cholesterol of 305 mg/dL and a triglyceride level of 142 mg/dL.

**Figure 2:**
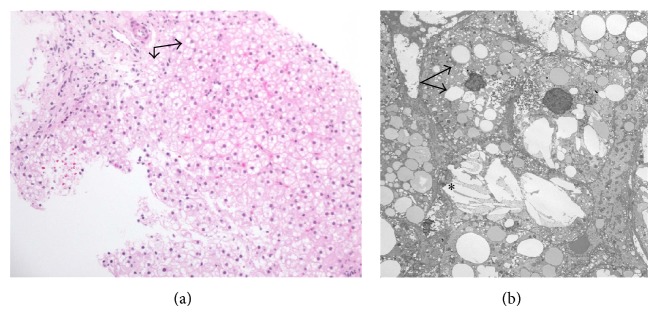
(a) Liver biopsy, periodic acid-Schiff stain with diastase (magnification, ×40). Lipid demonstrated within the hepatocellular cytoplasm (arrows). (b) Electron micrograph (direct magnification, ×2500). The hepatocellular cytoplasm in places has a moth-eaten appearance with lipid droplets (arrows). Additionally, some foci show cholesterol crystals (∗) free in the hepatocellular cytoplasm.

On the basis of a clinical suspicion of a diagnosis of CESD, a liver biopsy was performed. It revealed diffuse, microvesicular steatosis in the hepatic parenchyma (Figure 2(a)). Portal tracts were expanded by foamy macrophages containing finely vacuolated material, which was periodic acid-Schiff positive and resistant to diastase digestion. Trichrome stain for collagen showed mild portal fibrosis with early delicate bridging fibrosis. Ultrastructurally, the cytoplasm had a moth-eaten appearance with the presence of lipid droplets and cholesterol crystals (Figure 2(b)). There was no evidence of hepatocellular necrosis, cholestasis, or bile duct proliferation.

Liver tissue was sent to Dr. D. Wenger's Lysosomal Diseases Testing Laboratory at Thomas Jefferson University. She was found to have a low acid lipase activity of 7.2 (no units provided by Dr. D. Wenger's laboratory) in the liver. Follow-up testing through a commercial laboratory demonstrated low level of lysosomal acid lipase activity (0.008 nmol/punch/h) in blood. Lysosomal acid lipase gene sequence analysis was performed, which demonstrated that she was heterozygous for the following two mutations: c.57_60delTGAG and c.894G>A. Both parents underwent mutation analysis. The mother was confirmed to have the c.894G>A mutation while the father carried the c.57_60delTGAG mutation. The c.57_60delTGAG is a new mutation not previously described in the literature in patients with CESD.

## 3. Discussion

Cholesterol ester storage disease is an autosomal recessive, chronic liver disease caused by LAL deficiency. Its cognate gene is located on chromosome 10q23.3-q23.3 [2]. Lysosomal acid lipase hydrolyzes cholesterol esters and triglycerides that are delivered to the lysosomes by receptor-mediated endocytosis, and deficiency states result in accumulation of both cholesterol esters and triglycerides in hepatocytes [2]. Mutations in this gene cause two distinct phenotypes: WD and CESD. Both are characterized by hypercholesterolemia, hypertriglyceridemia, HDL deficiency, and hepatomegaly secondary to hepatic steatosis [2]. Complete absence of LAL activity results in WD, whereas CESD is due to mutations that result in partial loss of enzyme activity. Children diagnosed with CESD generally have a better prognosis but may often still require liver transplantation during their lifetime. They are also at risk for developing complications from cardiac disease.

Our patient's symptoms and presentation are typical of CESD. The histologic and ultrastructural examination of the liver along with markedly low LAL activity confirmed this diagnosis. DNA sequencing confirmed the presence of a novel hepatic mutation. It is a four-nucleotide deletion, c.57_60delTGAG in exon 2 of the LIPA gene. This mutation is predicted to result in a premature translation stop downstream of the deletion (p.E20fs) and, therefore, is felt to be a disease-causing mutation [4]. The second mutation noted in our patient is a previously described disease-causing mutation, c.894G>A change in exon 8 of the LIPA gene, which results in altered mRNA splicing and exon 8 skipping [5].

Current management is limited to preventing adverse effects of dyslipidemia. The use of statins [6, 7] and other cholesterol lowering agents such as cholestyramine and ezetimibe [1, 8, 9] has been associated with decreasing total cholesterol and increasing HDL, which is cardioprotective. Due to the young age of our patient, neither statins nor ezetimibe were prescribed for her hypercholesterolemia. In addition, lipid accumulation in the liver is not reversed with these medications. Patients may go on to develop cirrhosis and liver failure. Liver transplantation as a therapeutic option has been successfully used [1, 10, 11]. Direct enzyme replacement therapy with sebelipase alfa has been recently developed by Synageva BioPharma (Lexington, MA, USA) and is currently undergoing human trials [3, 12]. It has been recently reported in a phase 3 double blind placebo controlled trial that sebelipase alfa replacement enzyme therapy resulted in an improvement in ALT and AST with a relative reduction in hepatic fat fraction [13]. Thus, enzyme replacement therapy is a promising therapy that may change the long term outcome for patients with WD and CESD.
